# Crystal structure of bis­(2-amino-5-chloro­pyridinium) tetra­chlorido­cobaltate(II)

**DOI:** 10.1107/S2056989015007707

**Published:** 2015-04-25

**Authors:** Marwa Mghandef, Habib Boughzala

**Affiliations:** a Laboratoire de Matériaux et Cristallochimie, Faculté des Sciences de Tunis, Université de Tunis El Manar, 2092 Manar II Tunis, Tunisia

**Keywords:** crystal structure, cobalt(II) complex, 2-amino-5-chloro­pyridine, hydrogen bonding

## Abstract

The title salt, (C_5_H_6_ClN_2_)_2_[CoCl_4_], was synthesized by slow evaporation of an aqueous solution at room temperature. The asymmetric unit consists of two essentially planar (C_5_H_6_ClN_2_)^+^ cations [maximum deviations = 0.010 (3) and 0.014 (3) Å] that are nearly perpendicular to each other [dihedral angle = 84.12 (7)°]. They are bonded through N—H⋯Cl hydrogen bonds to distorted [CoCl_4_]^2−^ tetra­hedra, leading to the formation of undulating layers parallel to (100). The structure is isotypic with the Zn analogue [Kefi *et. al* (2011). *Acta Cryst.* E**67**, m355–m356.]

## Abstract 2

In the structure of bis­(2-amino-5-chloro­pyridinium) tetra­chlorido­cobaltate(II), the essentially planar cations are connected through N—H⋯Cl hydrogen bonds to the tetra­hedral anion.

## Chemical context   

Organic–inorganic hybrid compounds frequently exhibit self-organized structures and can combine organic and inorganic characteristics (Parent *et al.*, 2007 ▸; Zheng *et al.*, 2010 ▸; Chang *et al.*, 2011 ▸). In particular, anionic cobalt halides associated with organic counter-cations have some inter­esting physical properties, such as luminescence, in which we are inter­ested. In this communication, we report the synthesis and crystal structure of the new organic–inorganic hybrid compound bis­(2-amino-5-chloro­pyridinium) tetra­chlorido­cobaltate(II), (C_5_H_6_ClN_2_)_2_[CoCl_4_].
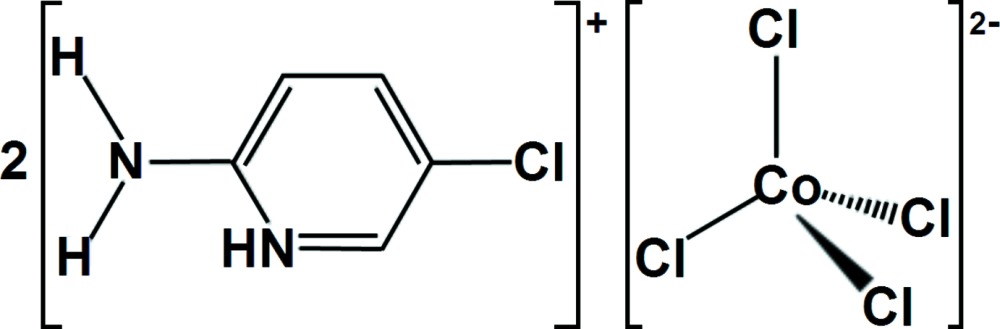


## Structural commentary   

**Figure 1:**
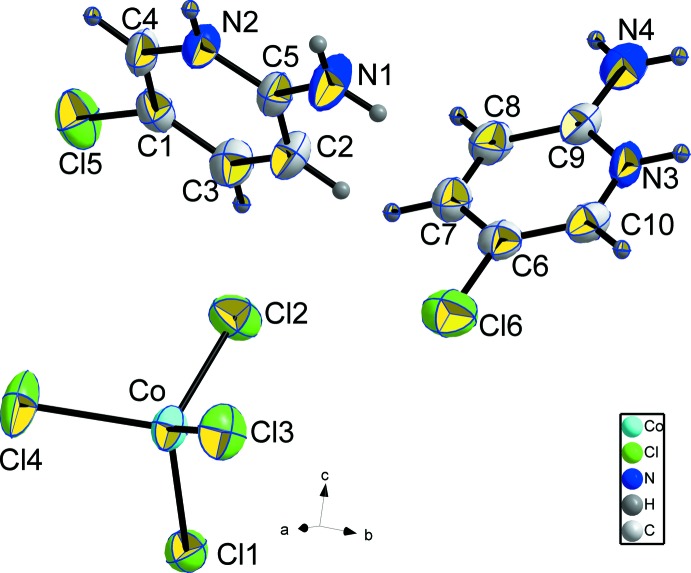
The mol­ecular entities of (C_5_H_6_ClN_2_)_2_[CoCl_4_], showing the atom-numbering scheme. Displacement ellipsoids are drawn at the 50% probability level.

The asymmetric unit of the title compound consists of two 2-amino-5-chloro­pyridinium cations (cat1 consists of ring C1–C5/N2 and cat2 consists of ring C9–C10/N3) and one isolated [CoCl_4_]^2−^ anion (Fig. 1 ▸).

The organic cations are nearly planar exhibiting small maximum deviations of 0.010 (3) and 0.014 (3) Å for atoms N2 and C6, respectively. The two least-squares planes of the two cations are nearly perpendicular to each other [84.12 (7)°]. The bond angles C4—N2—C5 [123.6 (3)°] and C9—N3—C10 [123.3 (3)°] in the rings of cat1 and cat2, respectively, confirm the presence of pyridinium cations. Previous studies (Jin *et al.*, 2001 ▸) showed that a pyridinium cation possesses an expanded C—N(H)—C angle in comparison with the parent pyridine (117°). This geometrical characteristic is in agreement with an imine–enamine resonance (Jin *et al.*, 2005 ▸) and contributes to the structural stability.

In the [CoCl_4_]^2−^ anion, the Co—Cl bond lengths range from 2.2645 (12) to 2.2934 (12) Å and the Cl—Co—Cl angles range from 104.84 (5) to 118.58 (5)°, revealing considerable distortions from the ideal tetra­hedral geometry. These values are in agreement with those observed in similar compounds (Dhieb *et al.*, 2014 ▸; Mghandef & Boughzala, 2014 ▸; Oh *et al.*, 2011 ▸). The different Co—Cl bond lengths in the [CoCl_4_]^2−^ anion are related to the number of hydrogen bonds accepted by the Cl atoms. The Co—Cl1 and Co—Cl4 bonds are longer than the Co—Cl2 and Co—Cl3 bonds because atoms Cl1 and Cl4 are each acceptors of two hydrogen bonds from cat2 and cat1, respectively.

## Supra­molecular features   

**Figure 2:**
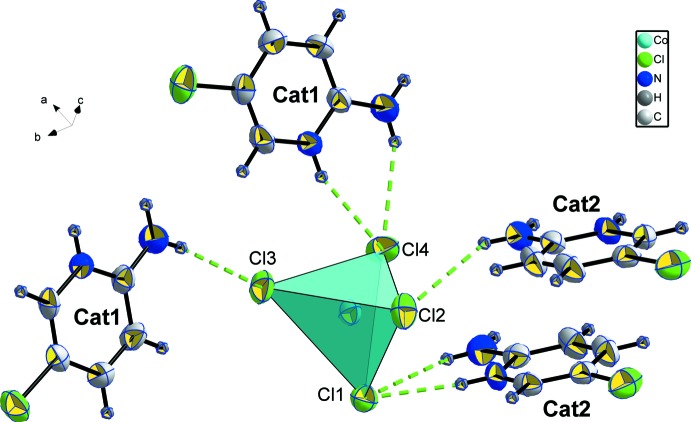
The environment of the CoCl_4_ tetra­hedron.

**Figure 3:**
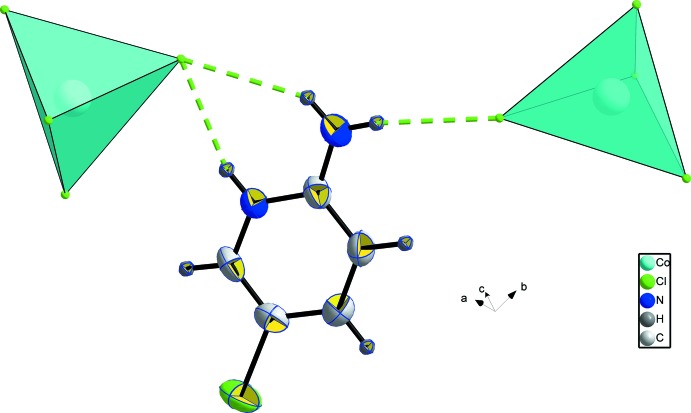
The environment around the cations (cat1 or cat2).

Each CoCl_4_ tetra­hedron is linked to four cations (two cat1 and two cat2) by hydrogen bonds (Fig. 2 ▸ and Table 1 ▸). Atom Cl1 is doubly linked to one cat2 cation by N3—H*N*3⋯Cl1 and N4—H4*A*⋯Cl1, and atom Cl2 establishes one hydrogen bond with a symmetry-related cat2 cation *via* N4—H4*B*⋯Cl2. Atom Cl3 is linked to cation cat1 by N1—H1*B*⋯Cl3 and atom Cl4 again shares two hydrogen bonds (N1—H1*A*⋯Cl4 and N2—H*N*2⋯Cl4) with a second symmetry-related cat1 cation. The hydrogen-bonding environments of the two cations are similar. Both are linked to two CoCl_4_ tetra­hedra by three hydrogen bonds (Fig. 3 ▸)

**Figure 4:**
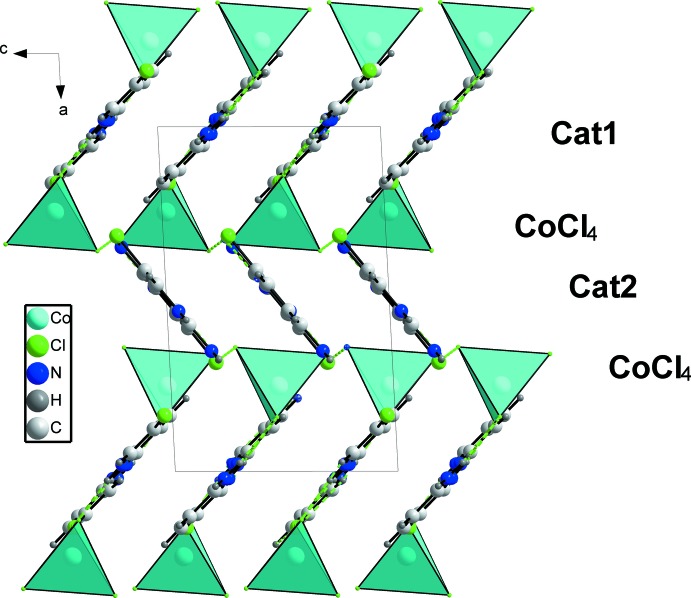
Projection of the crystal structure along [010] showing the –cat1–[CoCl_4_]–cat2–[CoCl_4_]– sequence stacked along [100].

**Figure 5:**
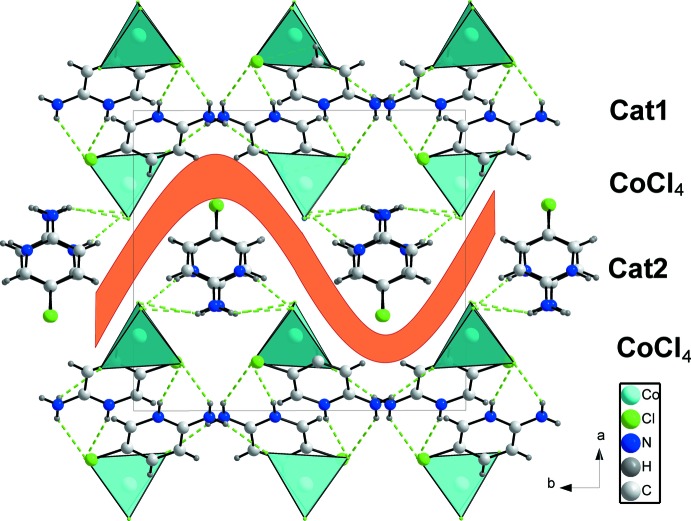
Projection of the crystal structure along [001] showing the layered character of the hydrogen-bonded components.

The crystal packing can be described by an alternate stacking of cations and anions with a –cat1–[CoCl_4_]–cat2–[CoCl_4_]– sequence along [100], as shown in Fig. 4 ▸. Between anti­parallel aligned cat2 cations, π–π inter­actions are also present [centroid-to-centroid separation = 3.900 (2) Å]. The stacked cations and anions are linked through N—H⋯Cl hydrogen bonds into zigzag layers parallel to (100) (Fig. 5 ▸).

## Database survey   

A systematic search procedure in the Cambridge Structural Database (Groom & Allen, 2014 ▸) indicates a total of 32 hits for the 2-amino-5-chloro­pyridinium cation with various counter-anions. For tetra­halogenidometalate anions, the following structures have been reported: (C_5_H_6_ClN_2_)_2_[ZnCl_4_]·H_2_O (Coomer *et al.*, 2007 ▸); (C_5_H_6_ClN_2_)_2_[ZnCl_4_] (Kefi *et al.*, 2011*a* ▸); (C_5_H_6_ClN_2_)_2_[CdCl_4_]·H_2_O (Kefi *et al.*, 2011*b* ▸); (C_5_H_6_ClN_2_)_2_[CuCl_4_] (Parsons *et al.*, 2006 ▸); (C_5_H_6_ClN_2_)_2_[CuBr_4_] (Woodward *et al.*, 2002 ▸). The title compound is isotypic with the Zn analogue (C_5_H_6_ClN_2_)_2_[ZnCl_4_] (Kefi *et al.*, 2011*a* ▸).

## Synthesis and crystallization   

A mixture of cobalt(II) chloride and 2-amino-5-chloro­pyridine (molar ratio 1:1) was dissolved in an aqueous solution of hydro­chloric acid with 5 ml of ethanol. The mixture was stirred and then kept at room temperature. Blue crystals of the title compound were obtained after two weeks.

## Refinement   

Crystal data, data collection and structure refinement details are summarized in Table 2 ▸. H atoms were placed geometrically and included as riding contributions, with N—H = 0.86 Å and C—H = 0.93 Å and with *U*_iso_(H) = 1.2*U*_eq_(N,C).

## Supplementary Material

Crystal structure: contains datablock(s) I, New_Global_Publ_Block. DOI: 10.1107/S2056989015007707/wm5146sup1.cif

Structure factors: contains datablock(s) I. DOI: 10.1107/S2056989015007707/wm5146Isup2.hkl

CCDC reference: 990478

Additional supporting information:  crystallographic information; 3D view; checkCIF report

## Figures and Tables

**Table 1:** Hydrogen-bond geometry (, )

*D*H*A*	*D*H	H*A*	*D* *A*	*D*H*A*
N1H1*A*Cl4^i^	0.86	2.64	3.400(4)	148
N1H1*B*Cl3^ii^	0.86	2.47	3.317(3)	169
N2H*N*2Cl4^i^	0.86	2.42	3.238(3)	160
N3H*N*3Cl1^iii^	0.86	2.42	3.251(3)	164
N4H4*A*Cl1^iii^	0.86	2.77	3.519(4)	147
N4H4*B*Cl2^iv^	0.86	2.80	3.541(4)	145

Symmetry codes: (i) 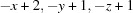; (ii) 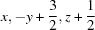; (iii) 
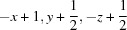
; (iv) 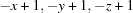.

**Table 2:** Experimental details

Crystal data	
Chemical formula	(C_5_H_6_ClN_2_)_2_[CoCl_4_]
*M*_r_	459.87
Crystal system, space group	Monoclinic, *P*2_1_/*c*
Temperature (K)	298
*a*, *b*, *c* ()	13.519(2), 14.945(3), 8.725(2)
()	92.858(3)
*V* (^3^)	1760.6(6)
*Z*	4
Radiation type	Mo *K*
(mm^1^)	1.88
Crystal size (mm)	0.5 0.3 0.2

Data collection	
Diffractometer	EnrafNonius CAD-4
Absorption correction	scan (North *et al.*, 1968 ▸)
*T*_min_, *T*_max_	0.423, 0.649
No. of measured, independent and observed [*I* > 2(*I*)] reflections	6241, 3707, 2121
*R*_int_	0.039
(sin /)_max_ (^1^)	0.638

Refinement	
*R*[*F* ^2^ > 2(*F* ^2^)], *wR*(*F* ^2^), *S*	0.041, 0.109, 0.99
No. of reflections	3707
No. of parameters	190
H-atom treatment	H-atom parameters not refined
_max_, _min_ (e ^3^)	0.52, 0.34

Computer programs: *CAD-4 EXPRESS* (EnrafNonius, 1994 ▸), *XCAD4* (Harms Wocadlo, 1995 ▸), *SHELXS97* and *SHELXL97* (Sheldrick, 2008 ▸), *DIAMOND* (Brandenburg, 2008 ▸) and *publCIF* (Westrip, 2010 ▸).

## supplementary crystallographic information

### Crystal data

**Table:**

(C_5_H_6_ClN_2_)_2_[CoCl_4_]	*F*(000) = 916
*M**_r_* = 459.87	*D*_x_ = 1.735 Mg m^−^^3^
Monoclinic, *P*2_1_/*c*	Mo *K*α radiation, λ = 0.71073 Å
Hall symbol: -P 2ybc	Cell parameters from 25 reflections
*a* = 13.519 (2) Å	θ = 10–15°
*b* = 14.945 (3) Å	µ = 1.88 mm^−^^1^
*c* = 8.725 (2) Å	*T* = 298 K
β = 92.858 (3)°	Prism, blue
*V* = 1760.6 (6) Å^3^	0.5 × 0.3 × 0.2 mm
*Z* = 4	

### Data collection

**Table:**

Enraf–Nonius CAD-4 diffractometer	2121 reflections with *I* > 2σ(*I*)
Radiation source: fine-focus sealed tube	*R*_int_ = 0.039
Graphite monochromator	θ_max_ = 27.0°, θ_min_ = 2.0°
non–profiled ω/2τ scans	*h* = −17→17
Absorption correction: ψ scan (North *et al.*, 1968)	*k* = −19→1
*T*_min_ = 0.423, *T*_max_ = 0.649	*l* = −11→5
6241 measured reflections	2 standard reflections every 120 min
3707 independent reflections	intensity decay: 6%

### Refinement

**Table:**

Refinement on *F*^2^	Primary atom site location: structure-invariant direct methods
Least-squares matrix: full	Secondary atom site location: difference Fourier map
*R*[*F*^2^ > 2σ(*F*^2^)] = 0.041	Hydrogen site location: inferred from neighbouring sites
*wR*(*F*^2^) = 0.109	H-atom parameters not refined
*S* = 0.99	*w* = 1/[σ^2^(*F*_o_^2^) + (0.0443*P*)^2^] where *P* = (*F*_o_^2^ + 2*F*_c_^2^)/3
3707 reflections	(Δ/σ)_max_ < 0.001
190 parameters	Δρ_max_ = 0.52 e Å^−^^3^
0 restraints	Δρ_min_ = −0.34 e Å^−^^3^

### Special details

**Table:**

Geometry. All e.s.d.'s (except the e.s.d. in the dihedral angle between two l.s. planes) are estimated using the full covariance matrix. The cell e.s.d.'s are taken into account individually in the estimation of e.s.d.'s in distances, angles and torsion angles; correlations between e.s.d.'s in cell parameters are only used when they are defined by crystal symmetry. An approximate (isotropic) treatment of cell e.s.d.'s is used for estimating e.s.d.'s involving l.s. planes.
Refinement. Refinement of *F*^2^ against ALL reflections. The weighted *R*-factor *wR* and goodness of fit *S* are based on *F*^2^, conventional *R*-factors *R* are based on *F*, with *F* set to zero for negative *F*^2^. The threshold expression of *F*^2^ > σ(*F*^2^) is used only for calculating *R*-factors(gt) *etc*. and is not relevant to the choice of reflections for refinement. *R*-factors based on *F*^2^ are statistically about twice as large as those based on *F*, and *R*- factors based on ALL data will be even larger.

### Fractional atomic coordinates and isotropic or equivalent isotropic displacement parameters (Å^2^)

**Table:**

	*x*	*y*	*z*	*U*_iso_*/*U*_eq_	
Co	0.75262 (3)	0.48488 (3)	0.01742 (6)	0.03927 (16)	
Cl1	0.66040 (7)	0.47789 (7)	−0.20888 (12)	0.0475 (3)	
Cl2	0.64020 (8)	0.48540 (8)	0.20135 (13)	0.0580 (3)	
Cl3	0.85951 (8)	0.60044 (7)	0.05556 (14)	0.0580 (3)	
Cl4	0.84305 (8)	0.35504 (7)	0.03059 (15)	0.0690 (4)	
Cl5	0.83772 (8)	0.37281 (8)	0.53616 (14)	0.0646 (3)	
Cl6	0.68358 (8)	0.75291 (9)	0.29194 (14)	0.0672 (4)	
N1	0.9792 (2)	0.7278 (2)	0.7188 (4)	0.0616 (11)	
H1A	1.0247	0.7306	0.7912	0.074*	
H1B	0.9565	0.7762	0.6768	0.074*	
N2	0.9798 (2)	0.5740 (2)	0.7372 (4)	0.0430 (8)	
HN2	1.0261	0.5790	0.8078	0.052*	
N3	0.4647 (2)	0.8197 (2)	0.5514 (4)	0.0444 (8)	
HN3	0.4394	0.8686	0.5834	0.053*	
N4	0.3491 (2)	0.7428 (2)	0.6902 (4)	0.0557 (9)	
H4A	0.3264	0.7931	0.7210	0.067*	
H4B	0.3233	0.6935	0.7195	0.067*	
C1	0.8770 (3)	0.4802 (3)	0.5868 (5)	0.0427 (9)	
C2	0.8694 (3)	0.6385 (3)	0.5537 (5)	0.0498 (11)	
H2	0.8425	0.6887	0.5045	0.060*	
C3	0.8370 (3)	0.5557 (3)	0.5125 (5)	0.0503 (11)	
H3	0.7880	0.5492	0.4346	0.060*	
C4	0.9472 (3)	0.4907 (2)	0.6989 (5)	0.0458 (10)	
H4	0.9736	0.4411	0.7504	0.055*	
C5	0.9440 (3)	0.6484 (2)	0.6714 (5)	0.0418 (9)	
C6	0.5837 (3)	0.7483 (3)	0.4092 (4)	0.0451 (10)	
C7	0.5437 (3)	0.6658 (3)	0.4521 (5)	0.0550 (11)	
H7	0.5707	0.6129	0.4168	0.066*	
C8	0.4669 (3)	0.6625 (3)	0.5436 (5)	0.0536 (11)	
H8	0.4413	0.6075	0.5720	0.064*	
C9	0.4253 (3)	0.7413 (3)	0.5959 (4)	0.0444 (10)	
C10	0.5418 (3)	0.8250 (3)	0.4591 (4)	0.0433 (9)	
H10	0.5659	0.8804	0.4301	0.052*	

### Atomic displacement parameters (Å^2^)

**Table:**

	*U*^11^	*U*^22^	*U*^33^	*U*^12^	*U*^13^	*U*^23^
Co	0.0394 (3)	0.0288 (3)	0.0492 (4)	−0.0023 (2)	−0.0018 (2)	−0.0013 (3)
Cl1	0.0502 (5)	0.0460 (6)	0.0459 (6)	0.0005 (5)	−0.0002 (4)	0.0005 (5)
Cl2	0.0679 (6)	0.0542 (7)	0.0534 (7)	−0.0144 (5)	0.0158 (5)	−0.0045 (6)
Cl3	0.0587 (6)	0.0409 (6)	0.0740 (8)	−0.0159 (5)	−0.0008 (5)	−0.0049 (6)
Cl4	0.0673 (7)	0.0336 (6)	0.1025 (10)	0.0110 (5)	−0.0328 (7)	−0.0098 (6)
Cl5	0.0688 (7)	0.0414 (6)	0.0842 (9)	−0.0114 (5)	0.0087 (6)	−0.0175 (6)
Cl6	0.0545 (6)	0.0911 (10)	0.0556 (7)	0.0035 (6)	−0.0007 (5)	0.0019 (7)
N1	0.072 (2)	0.031 (2)	0.079 (3)	−0.0005 (17)	−0.020 (2)	0.0032 (19)
N2	0.0468 (17)	0.0315 (18)	0.050 (2)	0.0029 (14)	−0.0070 (15)	0.0019 (16)
N3	0.058 (2)	0.0224 (16)	0.052 (2)	0.0068 (15)	−0.0028 (17)	−0.0015 (16)
N4	0.060 (2)	0.048 (2)	0.058 (2)	−0.0021 (17)	−0.0021 (19)	0.0081 (19)
C1	0.046 (2)	0.035 (2)	0.048 (2)	−0.0022 (18)	0.0102 (18)	−0.008 (2)
C2	0.049 (2)	0.041 (2)	0.058 (3)	0.0069 (19)	−0.009 (2)	0.010 (2)
C3	0.044 (2)	0.050 (3)	0.055 (3)	0.0022 (19)	−0.0083 (19)	−0.002 (2)
C4	0.054 (2)	0.025 (2)	0.058 (3)	0.0077 (17)	0.005 (2)	0.003 (2)
C5	0.044 (2)	0.031 (2)	0.051 (3)	0.0023 (17)	0.0027 (18)	0.006 (2)
C6	0.049 (2)	0.048 (3)	0.038 (2)	0.0016 (19)	−0.0102 (18)	−0.001 (2)
C7	0.082 (3)	0.035 (2)	0.048 (3)	0.007 (2)	−0.002 (2)	−0.001 (2)
C8	0.080 (3)	0.032 (2)	0.049 (3)	−0.006 (2)	0.003 (2)	0.005 (2)
C9	0.050 (2)	0.037 (2)	0.045 (2)	−0.0010 (18)	−0.0099 (19)	0.006 (2)
C10	0.049 (2)	0.037 (2)	0.043 (2)	−0.0078 (18)	−0.0065 (19)	0.0046 (19)

### Geometric parameters (Å, º)

**Table:**

Co—Cl2	2.2645 (12)	N4—H4A	0.8600
Co—Cl3	2.2657 (11)	N4—H4B	0.8600
Co—Cl1	2.2843 (12)	C1—C4	1.337 (5)
Co—Cl4	2.2934 (12)	C1—C3	1.397 (6)
Cl5—C1	1.741 (4)	C2—C3	1.355 (6)
Cl6—C6	1.735 (4)	C2—C5	1.410 (5)
N1—C5	1.337 (5)	C2—H2	0.9300
N1—H1A	0.8600	C3—H3	0.9300
N1—H1B	0.8600	C4—H4	0.9300
N2—C5	1.331 (4)	C6—C10	1.360 (5)
N2—C4	1.357 (5)	C6—C7	1.405 (6)
N2—HN2	0.8600	C7—C8	1.342 (6)
N3—C10	1.351 (5)	C7—H7	0.9300
N3—C9	1.352 (5)	C8—C9	1.392 (6)
N3—HN3	0.8600	C8—H8	0.9300
N4—C9	1.351 (5)	C10—H10	0.9300
			
Cl2—Co—Cl3	109.81 (5)	C2—C3—C1	120.1 (4)
Cl2—Co—Cl1	104.84 (5)	C2—C3—H3	119.9
Cl3—Co—Cl1	118.58 (5)	C1—C3—H3	119.9
Cl2—Co—Cl4	110.02 (5)	C1—C4—N2	119.9 (4)
Cl3—Co—Cl4	107.65 (5)	C1—C4—H4	120.1
Cl1—Co—Cl4	105.73 (4)	N2—C4—H4	120.1
C5—N1—H1A	120.0	N2—C5—N1	119.5 (3)
C5—N1—H1B	120.0	N2—C5—C2	117.2 (3)
H1A—N1—H1B	120.0	N1—C5—C2	123.3 (3)
C5—N2—C4	123.6 (3)	C10—C6—C7	118.8 (4)
C5—N2—HN2	118.2	C10—C6—Cl6	120.2 (3)
C4—N2—HN2	118.2	C7—C6—Cl6	120.9 (3)
C10—N3—C9	123.3 (3)	C8—C7—C6	120.7 (4)
C10—N3—HN3	118.4	C8—C7—H7	119.7
C9—N3—HN3	118.4	C6—C7—H7	119.7
C9—N4—H4A	120.0	C7—C8—C9	120.1 (4)
C9—N4—H4B	120.0	C7—C8—H8	120.0
H4A—N4—H4B	120.0	C9—C8—H8	120.0
C4—C1—C3	119.2 (4)	N4—C9—N3	118.9 (4)
C4—C1—Cl5	119.4 (3)	N4—C9—C8	123.1 (4)
C3—C1—Cl5	121.4 (3)	N3—C9—C8	117.9 (4)
C3—C2—C5	119.9 (4)	N3—C10—C6	119.2 (4)
C3—C2—H2	120.0	N3—C10—H10	120.4
C5—C2—H2	120.0	C6—C10—H10	120.4

### Hydrogen-bond geometry (Å, º)

**Table:**

*D*—H···*A*	*D*—H	H···*A*	*D*···*A*	*D*—H···*A*
N1—H1*A*···Cl4^i^	0.86	2.64	3.400 (4)	148
N1—H1*B*···Cl3^ii^	0.86	2.47	3.317 (3)	169
N2—H*N*2···Cl4^i^	0.86	2.42	3.238 (3)	160
N3—H*N*3···Cl1^iii^	0.86	2.42	3.251 (3)	164
N4—H4*A*···Cl1^iii^	0.86	2.77	3.519 (4)	147
N4—H4*B*···Cl2^iv^	0.86	2.80	3.541 (4)	145

Symmetry codes: (i) −*x*+2, −*y*+1, −*z*+1; (ii) *x*, −*y*+3/2, *z*+1/2; (iii) −*x*+1, *y*+1/2, −*z*+1/2; (iv) −*x*+1, −*y*+1, −*z*+1.
